# Supplementary material for: An experimental evaluation of an AI-powered interactive learning platform
Source: Front Artif Intell. 2026 Mar 10;9:1783117. doi: 10.3389/frai.2026.1783117 (PMC13008931; doi:10.3389/frai.2026.1783117)
Supplement: Supplementary file 1 [file Data_Sheet_1.zip › Supplementary Materials Frontiers in AI/Content Selection Criteria.pdf]

Content Selection Criteria

Through discussions with Pedagogy experts, we considered the following criteria in our approach for providing content that students will learn and be assessed on:

- **Motivation**: in order to incentivize learning, we'll prioritize topics that spark curiosity and have real-world application or relevance to the lives of participants (teenagers in Chicago).
- **Originality**: we are opting not to generate new or novel content to prevent the possibility of presenting information that might contradict with what participants have previously learned or encountered
- **Length**: we will prioritize content of shorter lengths to make it relatively easier to consume and optimize the learning time
- **Familiarity**: we will deprioritize content from the traditional US curriculum to reduce the likelihood of familiarity with the topic and maximize the learning experience for students.
- **Goal definition**: we will define learning objectives to guide the learning experience of students
- **Wellbeing**: we will do our best to control for content that is not inflammatory or triggering but we also give participants the option to discontinue participation if they feel uncomfortable at any point during the study.
- **Timing**: can be studied within the allotted learning time (ie 30 minutes)
- **Assessment**: has enough substance to inform the definition of learning objectives and questions for assessment
- **Format**: layout mimics what students are likely to receive in school or be assigned for reading. It can include some images but should not be an infographic.
