# Supplementary material for: An experimental evaluation of an AI-powered interactive learning platform
Source: Front Artif Intell. 2026 Mar 10;9:1783117. doi: 10.3389/frai.2026.1783117 (PMC13008931; doi:10.3389/frai.2026.1783117)
Supplement: Supplementary file 1 [file Data_Sheet_1.zip › Supplementary Materials Frontiers in AI/Long-Term Recall Assessment.pdf]

Long Term Recall Assessment (5-10 min)

[Show language]: Thank you again for your continued participation in this study. For this final survey, please **think back to the content you learned during your session** entitled, “Brain Development during Adolescence."

Remember to take your time and answer the following questions to the best of your ability. Please note, you **will not be able to go back and edit these responses** once you continue on to the next set of questions.

[NEW PAGE]

RA.Q1 During adolescence the brain undergoes significant structural and functional changes as it becomes more complex. Describe the relationship between these changes and common teen behaviors.

[Short answer]

____

[NEW PAGE]

RA.Q2 Which of the following options best describes the **changes that occur in the prefrontal cortex during adolescence?**

[Single select, Randomize]

- The structure of neurons in the prefrontal cortex changes leading to more efficient information processing and strengthened connections with other areas of the brain.
- The prefrontal cortex produces more neurotransmitters, which improves signal strength and communication speed between brain areas.
- Puberty hormones trigger the replacement of childhood neurons in the prefrontal cortex with adult neurons that process information at higher speeds and can connect to more neighboring neurons.

[NEW PAGE]

RA.Q3 Match the brain structure with its main function.

Please select only one response per row and column.

[Grid question]
[Columns]

- Prefrontal Cortex
- Limbic System

- Amygdala

[Rows, Randomize]

- Processes emotional and social experiences
- Executes cognitive functions such as decision-making
- Regulates the power of emotional responses
