# Supplementary material for: An experimental evaluation of an AI-powered interactive learning platform
Source: Front Artif Intell. 2026 Mar 10;9:1783117. doi: 10.3389/frai.2026.1783117 (PMC13008931; doi:10.3389/frai.2026.1783117)
Supplement: Supplementary file 1 [file Data_Sheet_1.zip › Supplementary Materials Frontiers in AI/Immediate Recall Scoring Rubric- SAQs.pdf]

Immediate Recall Scoring Rubric- SAQs

Base rubric for evaluation

Demonstrating (3 points)	Developing (2 points)	Emerging (1 point)	Fragmentary (0 points)
Response demonstrates a clear understanding of the concept.	Partial understanding of the concept is evident. Response contains a minor error or omission.	Limited understanding is evident, Response contains a substantial misconception or error.	Demonstrates a clear misunderstanding. Response is fragmentary or does not contain enough detail to demonstrate understanding.

Will not consider writing skills or grammar in evaluation

Immediate Recall Assessment

1. Give an example of common teen behavior that might be the result of developmental changes in adolescent brains. Explain the hypothesized relationship between the developmental changes and your example behavior.

	Demonstrating (3 points)	Developing (2 points)	Emerging (1 point)	Fragmentary (0 points)
Rubric	Response correctly connects a specific behavior to a developmental change, demonstrating a thorough understanding of the material. The change is described using specific details or scientific terminology.	Response correctly connects a specific behavior to a developmental change, demonstrating a basic understanding of the material. The change is described using general, non scientific terms. May confuse related scientific terms or lack clarity on the change, but core meaning is evident.	Response contains a misconception or omits an important aspect of the connection between common behaviors and developmental changes indicating that there is limited understanding of the material.	Response is fragmentary or completely lacking in detail. Answer may include phrases like “I don’t know” or “No clue.”

Model Answers	*(1) Teens struggle with impulsivity, this is because the pre frontal cortex isnt fully developed until mid to late twenties. another thing that is going on in the teenage brain is the hightened melatonin levels, making them constantly tired during the day and alert at night.*<br><br>*(2) more risk taking behaviors like drinking. this is because the part of the brain that controls pleasure is fully developed while the one controlling risk isnt*	*(1) An example of a common teen behavior due to brain developmental changes are staying up late at night and waking up later in the day due to the melatonin levels which increase within the blood at different rates and make teens come across as lazy. They can highten later at night because of the prefrontal cortex.*	*(1) doing risky things do to the prefrontal cortex being developed but the limbic part of the brain isn't. The prefrontal cortex makes things feel rewarding, like something telling you go, and the limbic says to stop, but since that part isn't fully developed, they don't get told to stop, and go through with whatever they were doing.*<br><br>*(2) A common teen behavior is mood changes/swings. I say this is a result of developmental changes because teens are still learning on how to act on their emotions and may not know when to stop because they are not thinking ahead of their further actions. The relationship is developing control your emotion and acting on them at the same time.*	*(1) teens are immature because their brain isn't really developed yet*<br><br>*(2) When the brain is developing it can have negative effects such as depreson.*
Score justification	(1) Correct connection, specific details<br><br>(2) Detailed description of the developmental	(1) Identifies a behavior and cause, describes in general terms, unclear how to interpret "They can highten later at night because of the	(1) Confuses function of prefrontal cortex and limbic system<br><br>(2) Alludes to changes, but ascribes to learning	Lacking in detail

	process	prefrontal cortex.”	rather than functional development.	

2. Teens are the target audience for social media apps such as TikTok. Use what you learned about teen brain development to explain the appeal of apps like TikTok to this audience.

	**Demonstrating (3 points)**	**Developing (2 points)**	**Emerging (1 point)**	**Fragmentary (0 points)**
Rubric	The response effectively explains the appeal of TikTok using specific developmental changes in adolescent brains.	The response explains the appeal of TikTok brain development using general terms. May confuse related scientific terms or lack clarity on the change, but core meaning is evident	The response omits important details in the explain or contains a misconception	Demonstrates a clear misunderstanding. Response is fragmentary or does not contain enough detail to demonstrate understanding.
Model Answer	*When teens see TikTok that excite them they continue to interact with it because of the limpic brain system. Since their brains are still developing they may not be able to get enough because they haven't gotten to the process that balances their excitement or plan to stop by the precortex system.*	*(1) teens are constantly seeking dopamine, tiktok gives that to us due to the short, intresting, infinate feed of videos. this stimulates our brain.*<br><br>*(2) TikTok gives people a good feeling do to the happy chemicals being produced, forgot what its called, just know its the other one that isn't serotonin or melatonin.*	*Tiktok reinforces social connections in the prefrontal cortex, making teens more likely to use it*	*teens like tiktok because they want to connect with their friends more than family*

Score justification	Specific detail on staggered function development	(1) Correct explanation, lack of detail connecting dopamine to scrolling<br><br>(2) Lite1 on detail	Misascribes social connection to pathways in the prefrontal cortex.	Does not connect appeal to brain development
