# Supplementary material for: An experimental evaluation of an AI-powered interactive learning platform
Source: Front Artif Intell. 2026 Mar 10;9:1783117. doi: 10.3389/frai.2026.1783117 (PMC13008931; doi:10.3389/frai.2026.1783117)
Supplement: Supplementary file 1 [file Data_Sheet_1.zip › Supplementary Materials Frontiers in AI/Immediate and Long Term Recall Assessment Design and Answers.pdf]

Immediate and Long Term Recall Assessment Design and Answers

Assessment Structure

- **Question type**: we will use a mix of questions types. Questions will be text dependent and not subjective. For example, “according to the text, when did xyz occur?”, “list X reasons why Y happened”, “what was the name of X in the text?”, “what were the arguments for or against X?” etc
- **Rubric constraint**: we will not evaluate writing skills as part of this assessment.
- **Number of questions**: it should be possible to answer all questions within 15 minutes
- **Generation process:** we prefer to have questions generated by humans to reduce the likelihood of prior exposure to students in the treatment group during quiz practice.

Proposed Additional Guidelines

- **Cognitive Complexity** Targeting specific levels of Bloom’s Taxonomy

Level	Description	Relevant action verbs
Recall	Recognizing or retrieving information from learned material	define, list, name, identify, state, label
Understand	Constructing meaning from learned material	explain, summarize, classify, discuss, restate
Apply	Applying learned material to new and concrete situations	use, demonstrate, solve, construct, choose
Analyze	Decomposing learned material into parts, exploring relationships between parts, and determining overall structure	compare, contrast, categorize, distinguish, infer
Evaluate	Assessing, making judgements, and drawing conclusions from learned material	rate, evaluate, assess, judge, justify
Synthesize	Combining elements of learned material into a new, coherent whole	create, compose, argue, support, revise, formulate

Development Process

Define Learning Objectives (Construct Identification)

- Describe brain development during adolescence
  - (Structure) Describe structural changes in the limbic system and prefrontal cortex.
  - (Function) Describe functional changes in the limbic system and prefrontal cortex.
  - (Milestones) Identify key milestones in brain development during adolescence.
  - (Behaviors) Explain the impact of developmental changes to common adolescent behaviors.

Question Formats

Short Answer Questions (SAQs)

- Written response 1 to 3 sentences in length
- Estimated response time of 3 to 5 mins
- Designed to gauge *understand, analyze, apply,* or *synthesize* levels
- Assessed on the following rubric

**Demonstrating (3 points)**	**Developing (2 points)**	**Emerging (1 point)**	**Fragmentary (0 points)**
Response demonstrates a clear understanding of the concept.	Partial understanding of the concept is evident. Response contains a minor error or omission.	Limited understanding is evident, Response contains a substantial misconception or error.	Demonstrates a clear misunderstanding. Response is fragmentary or does not contain enough detail to demonstrate understanding.

Single-Answer, Multiple Choice Questions (SMAQs)

- Single correct answer out of 3 or 4 choices
- Estimated response time of less than 1 min
- Designed to assess the recall, understand, or apply levels
- Assessed as correct (1 point) or incorrect (0 points)

Matching Questions

- Match 3 pairs of items

- Estimated response time of less than 1 min
- Designed to assess the recall or understand level
- Assessed on the following rubric

Fully Correct (2 points)	Partially Correct (1 point)	Incorrect (0 points)
All 3 pairs are correctly matched	1 pair is correctly matched	No pairs are correctly matched

Multi-Answer, Multiple Choice Questions (MMAQs)

- Multiple correct answers out of 3 or 4 choices
- Estimated response time of less than 2 mins
- Designed to assess the understand and apply levels
- Assessed on the following rubric

Fully Correct (2 points)	Partially Correct (1 point)	Incorrect (0 points)
All correctly answers are selected	At least one correct answer is not selected or a distractor is selected.	No correct answers are selected.

Post Learning Assessment

Assessment Outline

Question Type	Number of questions	Estimated time	Total possible points
**Short Answer**	2	8 mins	6
**Multiple Choice (single answer)**	2	2 mins	2
**Multiple Choice (multiple answer)**	1	2 mins	2
**Matching**	1	1 min	2
Total	7	13 mins	12

Preamble

Intent: Set the stage for assessment

[Draft some directions - Mirror language in the moderator guide]

Section 1: Short Answer Section

Intent: (1) Glean a summary of student learning in their own words; (2) Elicit the application of higher-order thinking skills; (3) Provide a basis for the retention assessment.

During adolescence the brain undergoes significant structural and functional changes as it becomes more complex.

1. Give an example of common teen behavior that might be the result of developmental changes in adolescent brains. Explain the hypothesized relationship between the developmental changes and your example behavior.

   Targeted skill(s): Recall and understand

   Model Answer: Teens are more likely to engage in risk-taking and thrill-seeking behavior, and not be deterred by the associated risks. This might be the result of the brain producing more dopamine, which helps experience pleasure and rewards, before the prefrontal cortex develops the ability to resist impulses.

2. Teens are the target audience for social media apps such as TikTok. Use what you learned about teen brain development to explain the appeal of apps like TikTok to this audience.

   Targeted skill(s): Apply and synthesize

   Model answer: TikTok highlights social and emotional information, which is processed by the limbic system. TikTok challenges offer social reward for risky behaviour, combining two traits of adolescent brains.

[After completing this section, students should not be allowed to return and revise their answers. This prevents students from using knowledge gleaned from later questions to answer earlier prompts.]

Section 2: Multiple Choice and Matching questions

Intent: Assess short-term recall and comprehension

Answer the following questions based on what you learned during this session.

3. Which of the following best describes the structural changes that occur in the brain's cortex during adolescence?
   a. The cortex produces new hormones which strengthen neural connections.
   b. The cortex grows new layers of neurons until it reaches adult size.
   c. **The cortex develops additional folds which increase the brain's complexity.**
   d. The cortex produces a new type of neural transmitter which improves processing.

Targeted skill(s): Recall

4. Match the brain chemical with its main function.

Targeted skill(s): Recall

dopamine	➢ controls the sleep-wake cycle<br>➢ **contributes to pleasure and decision-making**<br>➢ regulates of mood and behavior
serotonin	➢ controls the sleep-wake cycle<br>➢ contributes to pleasure and decision-making<br>➢ **regulates of mood and behavior**
melatonin	➢ **controls the sleep-wake cycle**<br>➢ contributes to pleasure and decision-making<br>➢ regulates of mood and behavior

5. Consider the following scenario: Alex is a high-school junior. He is still online playing video games with his friends at 1 am on a school night. He is annoyed when his dad makes him to turn-off the game and go to bed. While he knows he'll be groggy at school the next morning, he's not yet tired and wants to finish his quest with his friends before going to sleep. Which of the following developmental changes might explain Alex's reaction? *Choose all that apply.*

   a. Melatonin levels remain constant throughout the day and night during adolescence, which causes unpredictable sleep cycles.
   b. Adolescent melatonin levels naturally rise later at night and fall later in the morning, which delays the sleep cycle.

c. Increased activity in the amygdala results in a tendency to circumvent rules and dismiss authority figures.
d. The limbic system develops faster than the prefrontal cortex, which prioritises immediate rewards over long-term planning.

Targeted skill(s): Understand and apply

6. According to the text, which of the following best describes the final stage of adolescent brain development in early adulthood?
   a. The neural connections in the prefrontal cortex build more capacity to control cognition, leading to better judgment, more self-control, and improved long-term planning.
   b. The production of neurotransmitters by the limbic system stabilizes, leading to a reduction in irritability and risk-taking behaviors.
   c. Puberty hormones cease production, which calms the amygdala and reduces the perceived intensity of emotion and sensations.
   d. Neural pathways between the frontal lobe and other parts of the brain become bidirectional, increasing the brain's capacity to process social information.

Targeted skill(s): Understand

Retention Assessment

[Draft some directions - Mirror language in the moderator guide]

Assessment Outline

**Question Type**	**Number of questions**	**Estimated time**	**Points**
**Short Answer (SAQ)**	1	3 to 5 mins per SAQ ~ 3 min	3
**Multiple Choice (MCQ)**	1	1 min	1
**Matching**	1	1 min	2
Self-assessment	1	<1 min	NA
Total	4	6	6

Section 1: Short answer question

1. During adolescence the brain undergoes significant structural and functional changes as it becomes more complex. Describe the relations between these changes and common teen behaviors.

   Model Answer: The brain develops in stages during adolescence. The earlier stages are associated with intense feelings and sensations, but cognitive control does not fully develop until later stages. This makes teens more likely to have strong emotional responses and engage in risky behavior.

Targeted skill(s): Understand and analyze

[After completing this section, students should not be allowed to return and revise their answers. This prevents students from using knowledge gleaned from later questions to answer earlier prompts.]

Section 2: Multiple Choice and Matching Questions

2. Which of the following options best describe the changes that occur in the prefrontal cortex during adolescence?
   a. **The structure of neurons in the prefrontal cortex changes, leading to more efficient information processing and strengthened connections with other areas of the brain.**
   b. The prefrontal cortex produces more neurotransmitters, which improves signal strength and communication speed between brain areas.
   c. Puberty hormones trigger the replacement of childhood neurons in the prefrontal cortex with adult neurons that process information at higher speeds and can connect to more neighboring neurons.

Targeted skill(s): Understand

3. Match the brain region with its main function.

prefrontal cortex	➢ Processes emotional and social experiences<br>➢ **Executes cognitive functions such as decision-making**<br>➢ Regulates the power of emotional responses

limbic system	➢ **Processes emotional and social experiences**<br>➢ Executes cognitive functions such as decision-making<br>➢ Regulates the power of emotional responses
amygdala	➢ Processes emotional and social experiences<br>➢ Executes cognitive functions such as decision-making<br>➢ **Regulates the power of emotional responses**

Targeted skill(s): Understand and analyze

Section 3: Self Assessment

Intent: Assess student's confidence in their responses

1. How confident are you in the answers you provided during the assessment? [Likert Response]
    a. Not at all confident - I'm not sure about many of my answers
    b. Slightly confident - I'm not sure about some of my answers
    c. Somewhat confident - I feel good about most of my answers
    d. Very confident - I'm certain that all my answers are correct[Likert Response]
