# Supplementary material for: An experimental evaluation of an AI-powered interactive learning platform
Source: Front Artif Intell. 2026 Mar 10;9:1783117. doi: 10.3389/frai.2026.1783117 (PMC13008931; doi:10.3389/frai.2026.1783117)
Supplement: Supplementary file 1 [file Data_Sheet_1.zip › Supplementary Materials Frontiers in AI/Onboarding Slides_CHI (1).pdf]

Participant Onboarding

Digital Reader

Digital Reader

The PDF Reader allows you to read and annotate your reading material. It also allows you to view, read and print PDF files.

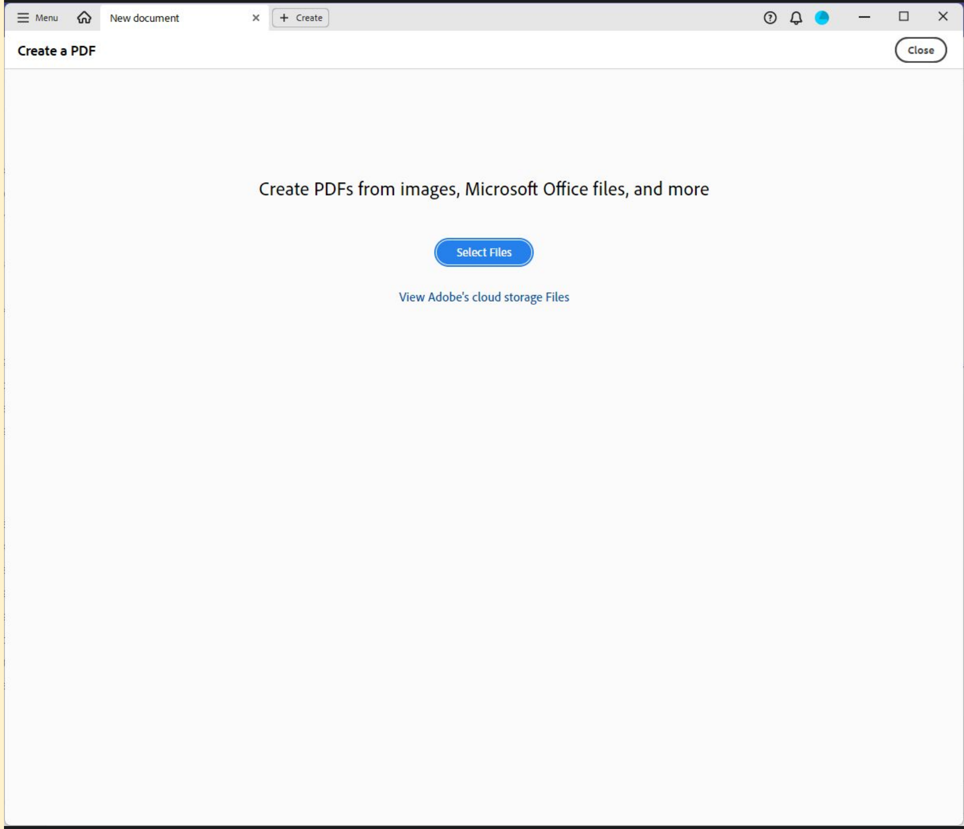

Getting Started

After opening the file, **you can use the features available in the PDF Reader to help with your learning journey**

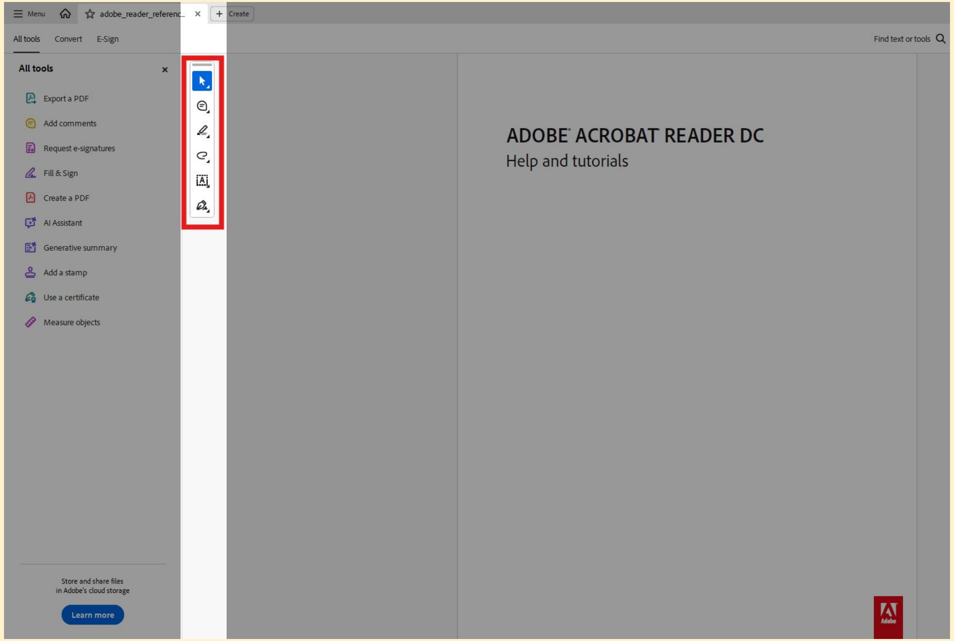

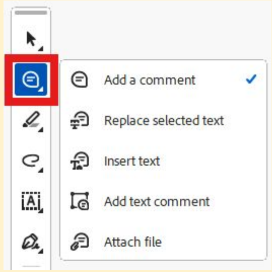

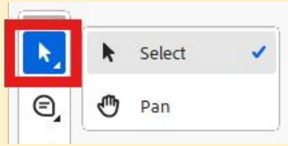

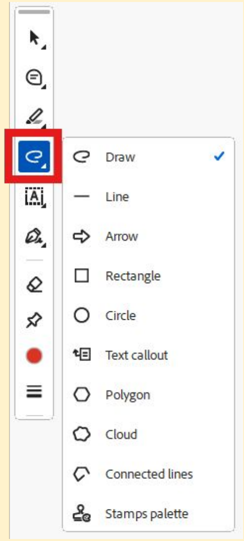

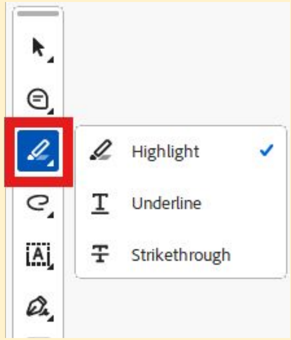

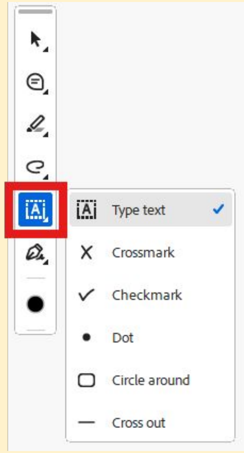

Remember that you are free to use any set of features within the PDF Reader to help you learn.

Hope you enjoy your learning journey!

Learn Your Way

Learn Your Way

Learn Your Way personalizes content based on your interests and learning goals. It also transforms content into multiple formats that you can choose from to learn your way.

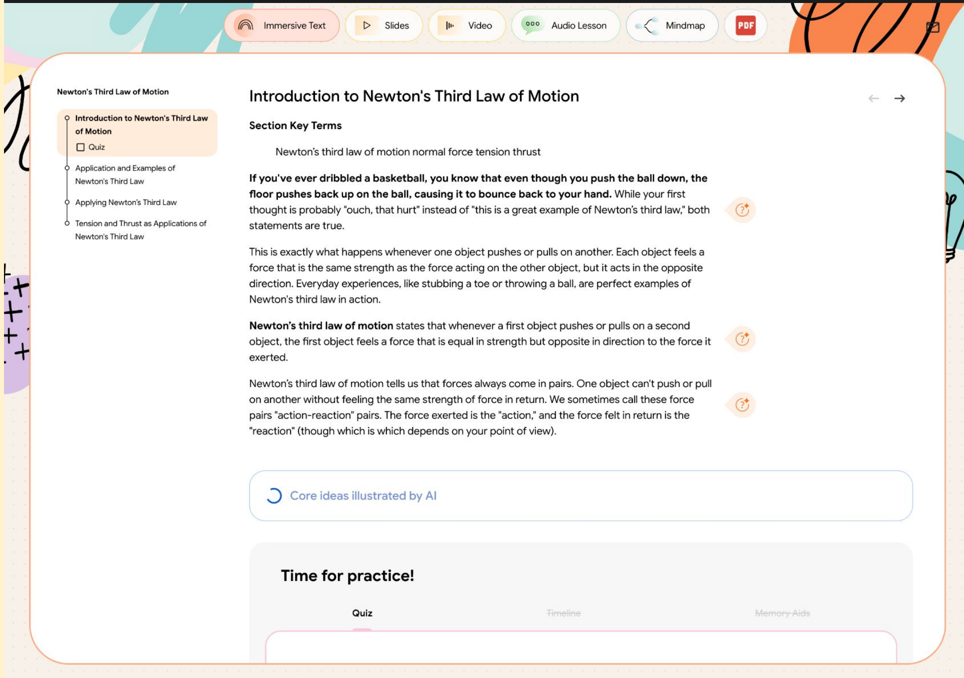

Getting Started

In Learn Your Way, **select the content you have been asked to learn today** from the bottom of the screen

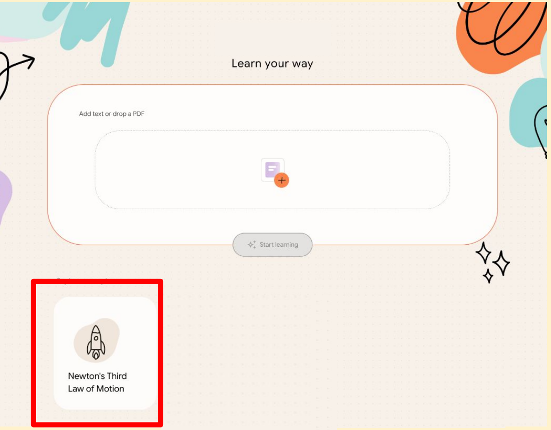

After selecting the content to learn, **you can personalize your learning experience** by selecting the **grade level you want to learn at** and **your interests**

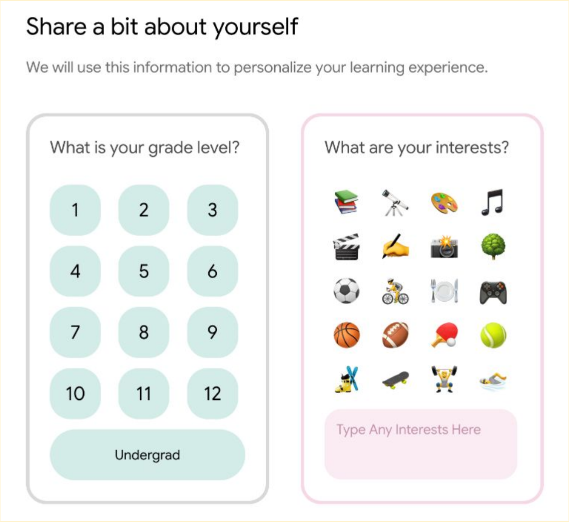

After selecting the content to learn, **you can use the features available in Learn Your Way to help with your learning journey**

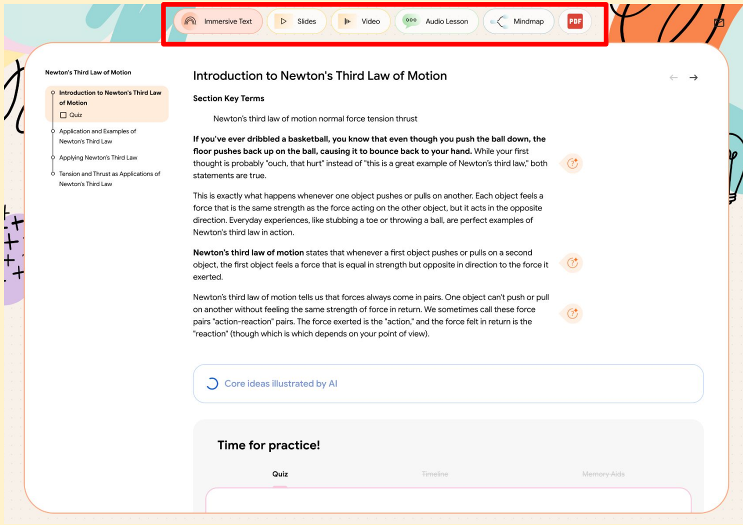

Remember that you are free to use any set of features within Learn Your Way to help you learn.

Hope you enjoy your learning journey!
