# Supplementary material for: An experimental evaluation of an AI-powered interactive learning platform
Source: Front Artif Intell. 2026 Mar 10;9:1783117. doi: 10.3389/frai.2026.1783117 (PMC13008931; doi:10.3389/frai.2026.1783117)
Supplement: Supplementary file 1 [file Data_Sheet_1.zip › Supplementary Materials Frontiers in AI/Long Term Recall Assessment- SAQ Scoring Rubric.pdf]

Long Term Recall Assessment- SAQ Scoring Rubric

1. During adolescence the brain undergoes significant structural and functional changes as it becomes more complex. Describe the relationship between these changes and common teen behaviors.

	**Demonstrating (3 points)**	**Developing (2 points)**	**Emerging (1 point)**	**Fragmentary (0 points)**
Rubric	Response correctly connects developmental changes to common teen behaviors, demonstrating a thorough understanding of the material by including specific details or scientific terminology.	Response correctly connects developmental changes to common teen behaviors, demonstrating a basic understanding of the material. The change is described using general, non scientific terms. May confuse related scientific terms or lack clarity on the change, but core meaning is evident.	Response contains a misconception or omits an important aspect of the connection between developmental changes and common teen behaviors, indicating that there is limited understanding of the material.	Response is fragmentary or completely lacking in detail. Answer may include phrases like “I don’t know” or “No clue.”
Model Answer	*The part of the brain responsible for pleasure is fully developed, while the part of the brain for risk and long term planning isn’t fully developed. This causes more dangerous behaviors*	*The part of the brain that tells someone to stop and think isn’t fully developed as a teen, so the reward part of the brain takes control and teens tend to do more risky behaviors*	*As the brain grows, teens become bored more easily. This is because the brain has more capacity.*	*As teens get older they become more mature.*
Score justification	Describes the staggered stages of system development and connects it to a common type of behavior.	Describes development in general terms, does not distinguish between stages	Confuses developmental changes with brain growth	Does not connect changes with brain development.
