# Supplementary material for: An experimental evaluation of an AI-powered interactive learning platform
Source: Front Artif Intell. 2026 Mar 10;9:1783117. doi: 10.3389/frai.2026.1783117 (PMC13008931; doi:10.3389/frai.2026.1783117)
Supplement: Supplementary file 1 [file Data_Sheet_1.zip › Supplementary Materials Frontiers in AI/Immediate Recall Assessment.pdf]

Immediate Recall Assessment

Thank you for taking the time to fill out this assessment on "Brain development during adolescence." We will start by having you **complete a couple short answer responses**.

Remember to take your time and answer the following questions to the best of your ability. Please note, you **will not be able to edit these responses** once you continue on to the next set of questions.

You will have up to **15 minutes to complete the full assessment**.

[NEW PAGE]

LA.Q1 Give an example of common teen behavior that might be the result of developmental changes in adolescent brains. Explain the hypothesized relationship between the developmental changes and your example behavior.

[Short answer]

____

LA.Q2 Teens are the target audience for social media apps such as TikTok. Use what you learned about teen brain development to explain the appeal of apps like TikTok to this audience.

[Short answer]

____

[NEW PAGE]

[Show language]: We would now like you to **answer the following multiple choice and matching questions** based on the content you read on "Brain development during adolescence."

LA.Q3 Which of the following best describes the **structural changes that occur in the brain's cortex during adolescence?**

[Single select, Randomize]

- The cortex produces new hormones which strengthen neural connections.
- The cortex grows new layers of neurons until it reaches adult size.
- The cortex develops additional folds which increase the brain's complexity.
- The cortex produces a new type of neural transmitter which improves processing.

[NEW PAGE]

LA.Q4 Match the brain chemical with its main function.

Please select only one response per row and column.

[Grid question]
[Columns]

- Dopamine
- Serotonin
- Melatonin

[Rows, Randomize]

- Controls the sleep-wake cycle
- Contributes to pleasure and decision-making
- Regulates mood and behavior

[NEW PAGE]

LA.Q5 Consider the following scenario: Alex is a high-school junior. He is still online playing video games with his friends at 1 am on a school night. He is annoyed when his dad makes him turn off the game and go to bed. While he knows he'll be groggy at school the next morning, he's not yet tired and wants to finish his quest with his friends before going to sleep.

Which of the following developmental changes best explains Alex's reaction?

Please select all that apply.

[Multi-select, Randomize]

- Melatonin levels remain constant throughout the day and night during adolescence, which causes unpredictable sleep cycles.
- Adolescent melatonin levels naturally rise later at night and fall later in the morning, which delays the sleep cycle.
- Increased activity in the amygdala results in a tendency to circumvent rules and dismiss authority figures.
- The limbic system develops faster than the prefrontal cortex, which prioritizes immediate rewards over long-term planning.

[NEW PAGE]

LA.Q6 According to the text, which of the following best describes the **final stage of adolescent brain development in early adulthood?**

[Single select, Randomize]

- The neural connections in the prefrontal cortex build more capacity to control cognition, leading to better judgment, more self-control, and improved long-term planning.
- The production of neurotransmitters by the limbic system stabilizes, leading to a reduction in irritability and risk-taking behaviors.
- Puberty hormones cease production, which calms the amygdala and reduces the perceived intensity of emotion and sensations.
- Neural pathways between the frontal lobe and other parts of the brain become bidirectional, increasing the brain's capacity to process social information.

[NEW PAGE]

LA.Q7 On a scale of 1-5, how **confident** are you in your responses to these assessment questions?

[Single select]

- 1 - Not at all confident
- 2
- 3
- 4
- 5 - Extremely confident
