# Supplementary material for: An experimental evaluation of an AI-powered interactive learning platform
Source: Front Artif Intell. 2026 Mar 10;9:1783117. doi: 10.3389/frai.2026.1783117 (PMC13008931; doi:10.3389/frai.2026.1783117)
Supplement: Supplementary file 1 [file Data_Sheet_1.zip › Supplementary Materials Frontiers in AI/Pre-Assessment Reading Comprehension Passage.pdf]

Pre-Assessment

The pre-assessment was given to all participants during recruitment to ensure students had similar levels of reading comprehension prior to the study. , The average score on the assessment was 6.4 out of 10, with a standard deviation of 2.3. We included students who scored 1 standard deviation above or below the mean, or those who scored a 4-9 out of 10 on the assessment

Passage

We would now ask you to read a short passage and answer a series of questions about the passage. This is intended to assess your reading skills. Read each passage and question carefully, and then choose the best answer to the question based on the passage.

All questions in this section are multiple-choice with four answer choices. Each question has a single best answer.

Please do not click out of the page at any time, or else you will be unable to participate in the research study.

**Passage**	**Questions**	**Answers**
This passage is adapted from Geoffrey Giller, "Long a Mystery, How 500-Meter-High Undersea Waves Form Is Revealed." ©2014 by Scientific American.<br><br>Some of the largest ocean waves in the world are nearly impossible to see. Unlike other large waves, these rollers, called internal waves, do not ride the ocean surface. Instead, they move underwater, undetectable without the use of satellite imagery or sophisticated monitoring equipment. Despite their	1. **The first paragraph serves mainly to**<br>A) explain how a scientific device is used.<br>B) note a common misconception about an event.<br>C) describe a natural phenomenon and address its importance.<br>D) present a recent study and summarize its findings.	**Choice C is the best answer**. In the first paragraph, the author identifies the natural phenomenon "internal waves" (line 3), and explains why they are important: "internal waves are fundamental parts of ocean water dynamics, transferring heat to the ocean depths and bringing up cold water from below" (lines 7-9).<br>Choices A, B, and D are incorrect because they do not identify the main purpose of the first paragraph, as that paragraph does not focus on a scientific device, a

hidden nature, internal waves are fundamental parts<br>of ocean water dynamics, transferring heat to the ocean depths and bringing up cold water from below. And they can reach staggering heights—some as tall<br>as skyscrapers.<br>Because these waves are involved in ocean mixing and thus the transfer of heat, understanding them is<br>crucial to global climate modeling, says Tom Peacock, a researcher at the Massachusetts Institute of Technology. Most models fail to take internal waves into account. “If we want to have more and more accurate climate models, we have to be able to<br>capture processes such as this,” Peacock says. Peacock and his colleagues tried to do just that.<br>Their study, published in November in Geophysical Research Letters, focused on internal waves generated<br>in the Luzon Strait, which separates Taiwan and the Philippines. Internal waves in this region, thought to be some of the largest in the world, can reach about 500 meters high. “That’s		common misconception, or a recent study.
	2. **As used in line 19, “capture” is closest in meaning to**<br>A) control.<br>B) record.<br>C) secure.<br>D) absorb	**Choice B is the best answer.** In lines 17-19, researcher Tom Peacock argues that in order to create precise global climate models, scientists must be able to “capture processes” such as how internal waves are formed. In this context, to “capture” a process means to record it for scientific study. Choices A, C, and D are incorrect because in this context “capture” does not mean to control, secure, or absorb.
	3. **According to Peacock, the ability to monitor internal waves is significant primarily because**<br>A) it will allow scientists to verify the maximum height of such waves.<br>B) it will allow researchers to shift their focus to improving the quality of satellite images.<br>C) the study of wave patterns will enable regions to predict and prevent coastal damage.<br>D) the study of such waves will inform the development of key scientific models.	**Choice D is the best answer.** In lines 17-19, researcher Tom Peacock argues that scientists need to “capture processes” of internal waves to develop “more and more accurate climate models.” Peacock is suggesting that studying internal waves will inform the development of scientific models. Choices A, B, and C are incorrect because Peacock does not state that monitoring internal waves will allow people to verify wave heights, improve satellite image quality, or prevent coastal damage
	4. **Which choice provides the best evidence for the answer to the previous question?**<br>A) Lines 1-2 (“Some... see”)<br>B) Lines 4-6 (“they... equipment”) C) Lines 17-19	**Choice C is the best answer.** In lines 17-19, researcher Tom Peacock provides evidence that studying internal waves will inform the development of key scientific models, such as “more accurate climate models.”

the same height as the Freedom Tower that's just been built in New York," Peacock says.<br>Although scientists knew of this phenomenon in<br>the South China Sea and beyond, they didn't know exactly how internal waves formed. To find out, Peacock and a team of researchers from M.I.T. and Woods Hole Oceanographic Institution worked with France's National Center for Scientific Research using a giant facility there called the Coriolis Platform. The rotating platform, about 15 meters (49.2 feet) in diameter, turns at variable speeds and<br>can simulate Earth's rotation. It also has walls, which<br>means scientists can fill it with water and create accurate, large-scale simulations of various oceanographic scenarios.<br>Peacock and his team built a carbon-fiber resin<br>scale model of the Luzon Strait, including the islands and surrounding ocean floor topography. Then they<br>filled the platform with water of varying salinity to replicate the different densities found at the strait,	("If... this")<br>D) Lines 24-26 ("Internal... high")	Choices A, B, and D are incorrect because they do not provide the best evidence that studying internal waves will inform the development of key scientific models; rather, they provide general information about internal waves.
	5. **As used in line 65, "devise" most nearly means**<br>A) create.<br>B) solve.<br>C) imagine.<br>D) begin	**Choice A is the best answer.**<br>In lines 65-67, the author notes that Tom Peacock and his team "were able to devise a mathematical model that describes the movement and formation of these waves." In this context, the researchers devised, or created, a mathematical model. Choices B, C, and D are incorrect because in this context "devise" does not mean to solve, imagine, or begin.
	6. **Based on information in the passage, it can reasonably be inferred that all internal waves**<br>A) reach approximately the same height even though the locations and depths of continental shelves vary.<br>B) may be caused by similar factors but are influenced by the distinct topographies of different regions.<br>C) can be traced to inconsistencies in the tidal patterns of deep ocean water located near islands.<br>D) are generated by the movement of dense water over a relatively flat section of	**Choice B is the best answer.**<br>Tom Peacock and his team created a model of the "Luzon's Strait's underwater topography" and determined that its "distinct double-ridge shape . . . [is] responsible for generating the underwater [internal] waves" (lines 53-55). The author notes that this model describes only internal waves in the Luzon Strait but that the team's findings may "help researchers understand how internal waves are generated in other places around the world" (lines 67-70). The author's claim suggests that while internal waves in the Luzon Strait are "some of the

with denser, saltier water below and lighter, less briny water above. Small particles were added to the solution and illuminated with lights from below in order to track how the liquid moved. Finally, they re-created tides using two large plungers to see how the internal waves themselves formed.<br>The Luzon Strait's underwater topography, with a<br>distinct double-ridge shape, turns out to be responsible for generating the underwater waves.<br>As the tide rises and falls and water moves through the strait, colder, denser water is pushed up over the<br>ridges into warmer, less dense layers above it.<br>This action results in bumps of colder water trailed<br>by warmer water that generate an internal wave.<br>As these waves move toward land, they become steeper—much the same way waves at the beach become taller before they hit the shore—until they break on a continental shelf.<br>The researchers were also able to devise a mathematical model that describes the movement	the ocean floor	largest in the world" (line 25) due to the region's topography, internal waves occurring in other regions may be caused by some similar factors. Choice A is incorrect because the author notes that the internal waves in the Luzon Strait are "some of the largest in the world" (line 25), which suggests that internal waves reach varying heights. Choices C and D are incorrect because they are not supported by the researchers' findings.
	**7. Which choice provides the best evidence for the answer to the previous question?**<br>A) Lines 29-31 ("Although... formed")<br>B) Lines 56-58 ("As the... it")<br>C) Lines 61-64 ("As these... shelf")<br>D) Lines 67-70 ("Whereas... world")	**Choice D is the best answer.**<br>In lines 67-70, the author provides evidence that, while the researchers' findings suggest the internal waves in the Luzon Strait are influenced by the region's topography, the findings may "help researchers understand how internal waves are generated in other places around the world." This statement suggests that all internal waves may be caused by some similar factors. Choices A, B, and C are incorrect because they do not provide the best evidence that internal waves are caused by similar factors but influenced by the distinct topographies of different regions. Rather, choices A, B, and C reference general information about internal waves or focus solely on those that occur in the Luzon Strait.
	**8. In the graph, which**	**Choice D is the best answer.**

<table>
<tr>
<td rowspan="3">and formation of these waves. Whereas the model is
specific to the Luzon Strait, it can still help researchers understand how internal waves are generated in other places around the world. Eventually, this information will be incorporated into global climate models, making them more accurate.
“It’s very clear, within the context of these [global climate] models, that internal waves play a role in
driving ocean circulations,” Peacock says.
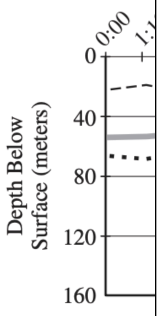

Adapted from Justin Small et al., “Interna</td>
<td>isotherm displays an increase in depth below the surface during the period 19:12 to 20:24?
A) 9°C
B) 10°C
C) 11°C
D) 13°C</td>
<td>During the period 19:12 to 20:24, the graph shows the 13°C isotherm increasing in depth from about 20 to 40 meters. Choices A, B, and C are incorrect because during the time period 19:12 to 20:24 the 9°C, 10°C, and 11°C isotherms all decreased in depth.</td>
</tr>
<tr>
<td>9. Which concept is supported by the passage and by the information in the graph?
A) Internal waves cause water of varying salinity to mix.
B) Internal waves push denser water above layers of less dense water.
C) Internal waves push bands of cold water above bands of warmer water.
D) Internal waves do not rise to break the ocean’s surface</td>
<td>Choice D is the best answer.
In lines 3-6, the author notes that internal waves “do not ride the ocean surface” but “move underwater, undetectable without the use of satellite imagery or sophisticated monitoring equipment.” The graph shows that the isotherms in an internal wave never reach the ocean’s surface, as the isotherms do not record a depth of 0. Choice A is incorrect because the graph provides no information about salinity. Choice B is incorrect because the graph shows layers of less dense water (which, based on the passage, are warmer) riding above layers of denser water (which, based on the passage, are cooler). Choice C is incorrect because the graph shows that internal waves push isotherms of warmer water above bands of colder water.</td>
</tr>
<tr>
<td>10. How does the graph support the author’s point that internal waves affect ocean water dynamics?
A) It demonstrates that wave</td>
<td>Choice A is the best answer.
In lines 7-9, the author notes that internal waves are “fundamental parts of ocean water dynamics” because they transfer “heat to the ocean</td>
</tr>
</table>

	movement forces warmer water down to depths that typically are colder.<br>B) It reveals the degree to which an internal wave affects the density of deep layers of cold water.<br>C) It illustrates the change in surface temperature that takes place during an isolated series of deep waves.<br>D) It shows that multiple waves rising near the surface of the ocean disrupt the flow of normal tides.	depths and bring up cold water from below.” The graph shows an internal wave forcing the warm isotherms to depths that typically are colder. For example, at 13:12, the internal wave transfers “heat to the ocean depths” by forcing the 10°C, 11°C, and 13°C isotherms to depths that typically are colder. Choices B, C, and D are incorrect because the graph does not show how internal waves affect the ocean’s density, surface temperature, or tide flow.
