# Supplementary material for: An experimental evaluation of an AI-powered interactive learning platform
Source: Front Artif Intell. 2026 Mar 10;9:1783117. doi: 10.3389/frai.2026.1783117 (PMC13008931; doi:10.3389/frai.2026.1783117)
Supplement: Supplementary file 1 [file Data_Sheet_1.zip › Supplementary Materials Frontiers in AI/Immediate Recall Assessment- Learning Experience Metrics.pdf]

Immediate Recall Assessment: Learning Experience Metrics (5-10 min)

[Show language]: Thank you for taking your time to complete this assessment.

We would now like to have you complete a brief survey about your **familiarity with the content** and your **experience with the educational tool** you just used to learn the content on “Brain Development during Adolescence" today.

You will have **5-10 minutes to complete the following survey.**

[NEW PAGE]

S.Q1 Prior to today's session, how **familiar** were you with the content on “Brain Development during Adolescence”?

[Single select]

- Not at all familiar
- Slightly familiar
- Somewhat familiar
- Very familiar

S.Q2 And how **interesting** did you find the content on “Brain Development during Adolescence”?

[Single select]

- Not at all interesting
- Slightly interesting
- Somewhat interesting
- Very interesting

S.Q3 How **easy** or **difficult** was the assessment for you?

[Single select]

- Very difficult
- Somewhat difficult
- Neither difficult nor easy
- Somewhat easy
- Very easy

[NEW PAGE]

S.Q4 How **easy** or **difficult** was it for you to use the educational tool today?

[Single select]

- Very difficult
- Somewhat difficult
- Neither difficult nor easy
- Somewhat easy
- Very easy

S.Q5 How **useful** was the educational tool in helping you learn the content on “Brain Development during Adolescence?”

[Single select]

- Not at all useful
- Slightly useful
- Somewhat useful
- Very useful

[NEW PAGE]

S.Q6 To what extent do you **agree** or **disagree** with the following statements?

[Carousel question; Single response per row]
[Columns]

- Strongly disagree
- Somewhat disagree
- Neither agree nor disagree
- Somewhat agree
- Strongly agree

[Rows, Randomize]

- The educational tool I used today would make me more effective at learning compared to other educational tools I currently use at home or in school.
- I would like to use today’s educational tool to support my learning needs in the future.
- I felt like I performed well on the assessment.
- I felt like today’s educational tool made me more comfortable taking the assessment.
- I found today’s educational tool enjoyable to use.
- I would recommend this educational tool to other students to support their learning needs.
- I felt like today's educational tool helped me gain a good understanding of the content.
