# Supplementary material for: An experimental evaluation of an AI-powered interactive learning platform
Source: Front Artif Intell. 2026 Mar 10;9:1783117. doi: 10.3389/frai.2026.1783117 (PMC13008931; doi:10.3389/frai.2026.1783117)

Learn Your Way Modalities (Visual Example)

# Immersive Text

Immersive Text, which includes Section-level quizzes (QuizMe), Embedded questions, AI-Generated Images and Summary (Enimate), Timeline, Memory Aids

**
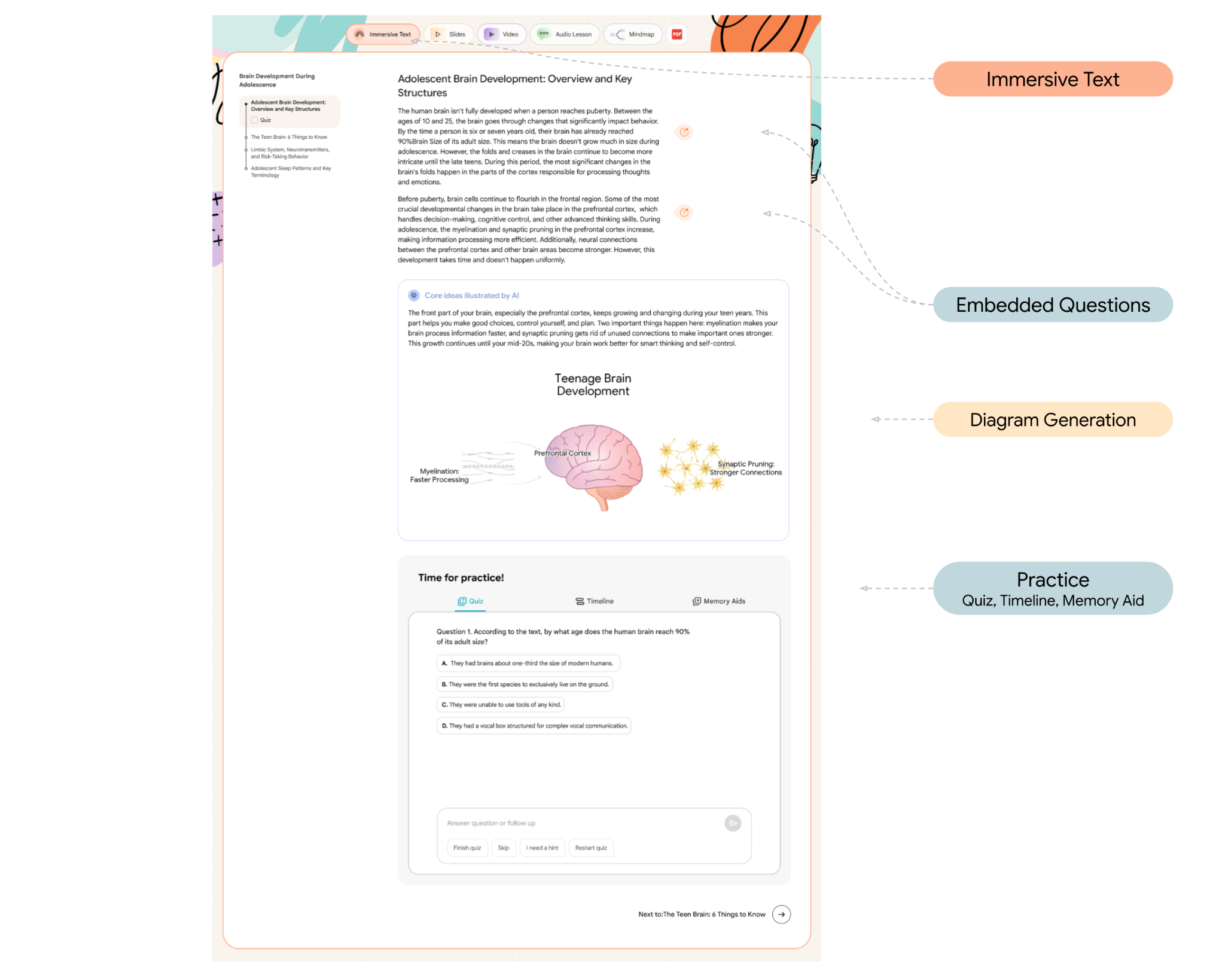
**

# Slides

**
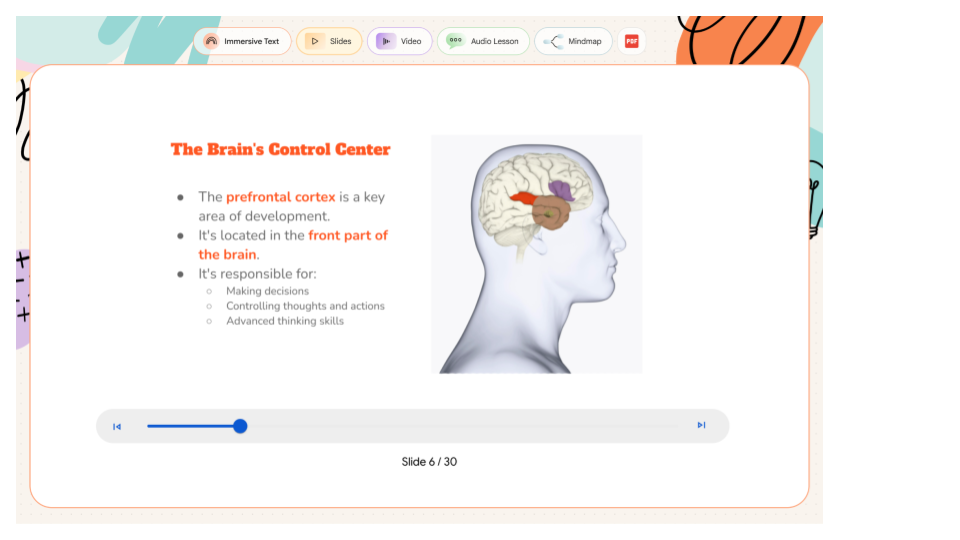
**

# Video (slide deck with narration):


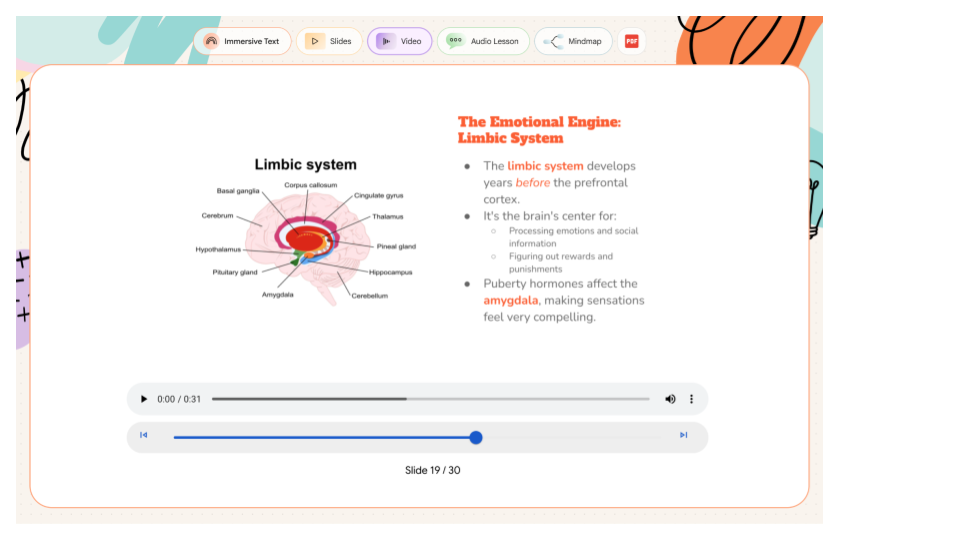


# Audio Lessons

**
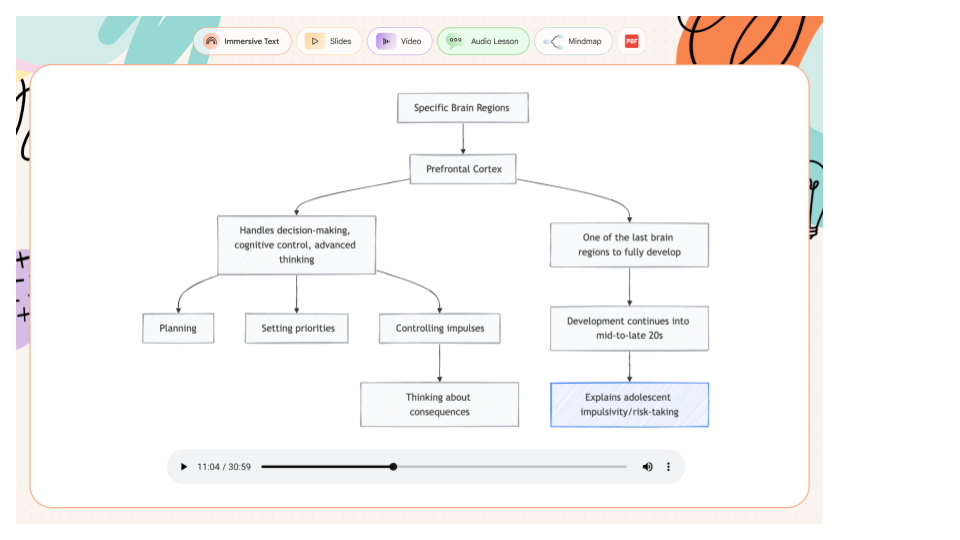
**

# Mindmap


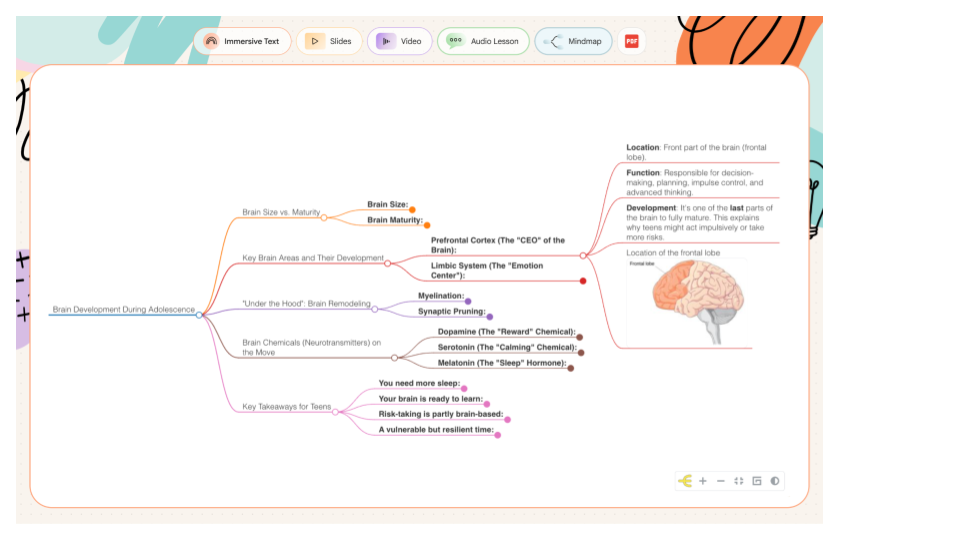

Supplement: Supplementary file 1 [file Data_Sheet_1.zip › Supplementary Materials Frontiers in AI/Learn Your Way Modalities.docx]
