# Supplementary material for: An experimental evaluation of an AI-powered interactive learning platform
Source: Front Artif Intell. 2026 Mar 10;9:1783117. doi: 10.3389/frai.2026.1783117 (PMC13008931; doi:10.3389/frai.2026.1783117)
Supplement: Supplementary file 1 [file Data_Sheet_1.zip › Supplementary Materials Frontiers in AI/Rubrics for Pedagogical Evaluations + Results.docx]

## **Rubrics for Pedagogical Evaluations + Results**

Pedagogical rubrics used by experts to rate the various components of \textit{Learn Your Way}*.*

*Raters were also given the option of marking N/A or can’t assess for each dimension.*

| DIMENSION | DESCRIPTION | AGREE (1) | NEUTRAL/PARTIAL (0.5) | DISAGREE (0) |
| --- | --- | --- | --- | --- |
| Accuracy | Faithful to the source and accurate | The generated content is consistent with the source resource. Does not misrepresent, but miscorrects concepts. Captures but clearly conveys the source’s main arguments, supporting key claims, and presentation of the content (when applicable) is accurate and factual. | Content does not add or alter main concepts, but may have minor inaccuracies, missing concepts, relationships, or arguments. | Content contains claims or arguments that counter the source, major inaccuracies, or misses major concepts. |
| Coverage | Completeness of representation of source messages | The generated content covers the source content, faithfully preserving the links and structure of the material. The source hierarchy is preserved. All high- and low-level concepts and LOs are covered, but immaterial details may be omitted to support learnability. For global constructs, global coverage has local constructs - local coverage i.e. projected to the scope and context of content. | Content mostly covers the source and subject matter of the source, but is missing some key concepts, relationships, or arguments. Source hierarchy is not preserved, some immaterial details are carried over while more important elements omitted. | Key subject matter or structure is missing from the generated content. |
| Emphasis | Precisely prioritizes core concepts. Idealizes the content hierarchy and optimal choices and places key messages. | The generated content focuses on the key concepts / relationships in the source, as crucial / essential / exciting content. For global constructs: The hierarchy of elements coincides with the knowledge / skills hierarchy of the source. This is consistent with the generated content focuses on the prominent elements in the source. | Choice of elements partially reflects source hierarchy or intentions by including some of the core concepts but might miss an important concept or emphasis some trivial material along with the core concepts. | Choice of elements appears arbitrary, and does not align with source hierarchy or intent. Generated content highlights immaterial concepts or relationships instead of key messages. |
| Engagement | Positive, pleasant and purposeful user experience | The generated content evokes positive emotional response. The tone, aesthetics, narrative, presentation, and elements of the content create a fun and captivating learning experience. | Content does not consistently engage or evoke a positive response from the user. Limited use of playfulness or personalization. Utility and relevance to user is present, but might not be clear to the user. | Content is dry, boring, does not attempt to evoke positive emotions. Monotonous single-track presentation. No perceived value to the user. |
| Cognitive Load | Effective use of cognitive effort | Content is well organized, clear and concise. Accessible language. Key points emphasized. Uses metaphors, analogies, narratives, and multiple representations effectively to enhance understanding. | Content is fairly well structured but some sections could be improved. Repetitive, suboptimal formats. Some unhelpful redundancy. Limited use of examples, analogies, narratives, or multiple representations to enhance learning. | Content is poorly structured, and may include large overwhelming blocks of text. No clear hierarchy. The learning level is inconsistent, incorporating difficult concepts, oversimplifying for the given level. No use of aids such as examples, analogies, narratives, or multiple representations. |
| Active Learning | Promotes active learning that goes beyond recall and memorization. | Content raises questions and encourages user to engage with subject matter and learning objectives. It includes recalls, recaps, comprehension, analysis, application, evaluation, and creation. | Content mostly focuses on information delivery, and misses opportunities to engage with learning objectives at a deeper level. | Content is mostly informational and does not encourage higher order thinking. |
| Deep Metacognition | Promotes active self-reflection, monitoring, regulation. Use, improvement of thinking and learning processes. | Promotes relevant and actionable feedback to improve active reflection and inspection of learning processes. Supports the user to identify, execute and monitor their learning plan. | Provides somewhat useful feedback, but may not be relevant or actionable. Prompts shallow reflection without encouraging self-regulation. Does not provide consideration of learning plans. | Flat informative content, no useful feedback, no prompts for reflection or managing and regulating learning. |
| Motivation & Curiosity | Encourages user to persist and deeply engage with learning processes. Incentivizes cognitive effort. | Uses a consistently supportive tone, encouraging user to persist. Maintains an optimal level of challenge, between too easy (boring) and too hard (frustrating). Highlights the relevance of the subject matter to the user, their concerns and interests. Employs cues and autonomy to make a variety of viable paths of learning depth, pace, order and focus. | Tone is occasional unsupportive, or encouragement is sporadic. Challenges are not consistently aligned to an optimal challenge and/or interest. Limited use of examples and real-life connections to highlight subject matter relevance to user's concerns and interests. User choices in the learning process are not evident. | Tone is not supportive or encouraging. Challenges are missing, or at an inappropriate level for the user. No evident links from the subject matter to user concerns and interests. No choice in learning path. |
| Adaptability & Personalization | Learning experience is adjusted to fit the user interests, preferences and characteristics. | Content is fully adjusted to user's interests, age group, grade level, language, proficiency level, literacy, prior knowledge, goals, and preferences. Challenges are appropriate for the user's capabilities and interests. | Content acknowledges user's preferences and characteristics, but is only partially adjusted. Challenges are not consistently aligned with user capability or interest. | Content ignores user preferences and characteristics, "one size fits all". |
| Clarity of Learning Intentions & Success Criteria | Clearly articulates what is being learned (learning objectives/goals/objectives) and how users will know if they have been successful (success criteria). | Learning objectives are clearly stated and user-friendly. Specific rubrics or benchmarks define what success is completely, or under what learning state. Consistent with LOs, with stated LOs and criteria (if provided). | Learning objectives stated but not elaborated or demonstrated. Success criteria are vague or confusing for the user. Learning objectives with LOs partial or unintuitive (if provided). | Learning objectives not specified, unclear or inconsistent. No evident success criteria. Content misaligned with LOs (if provided). |

### **Learn Your Way Evaluation Results**

For each capability, three expert pedagogical raters, selected for their expertise in both the target grade level and subject matter, evaluated the generated content. This evaluation utilized 10 distinct source materials from existing textbooks, spanning various subjects from history to physics. Each source material was tested across three different personalization configurations, resulting in 30 unique configurations for evaluation.

A sample of these results is provided for reference. This expert feedback was instrumental in measuring and refining the quality of each component.

*
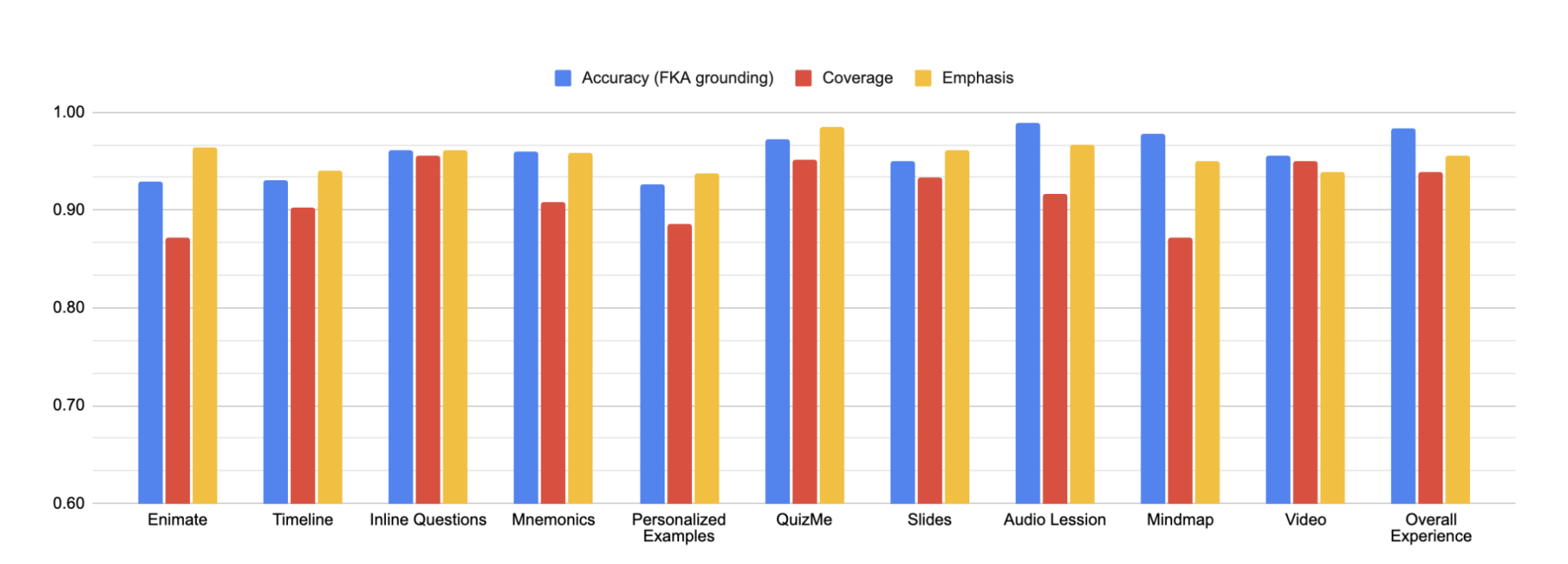
*

Caption: Average ratings from pedagogy experts evaluating \textit{Learn Your Way’s} capabilities across accuracy, coverage, and emphasis.

AD: This is a **bar chart** that compares different educational tools across three evaluation metrics: **Accuracy**, **Coverage**, and **Emphasis**. The chart's x-axis lists the educational tools, and the y-axis represents a score from **0.60 to 1.00**.

### **Educational Tools and Their Scores**

The chart displays data for the following tools, from left to right:

- **Enimate**:
  - Accuracy (blue bar): Approximately 0.93
  - Coverage (red bar): Approximately 0.87
  - Emphasis (yellow bar): Approximately 0.96
- **Timeline**:
  - Accuracy (blue bar): Approximately 0.93
  - Coverage (red bar): Approximately 0.90
  - Emphasis (yellow bar): Approximately 0.94
- **Inline Questions**:
  - Accuracy (blue bar): Approximately 0.96
  - Coverage (red bar): Approximately 0.96
  - Emphasis (yellow bar): Approximately 0.96
- **Mnemonics**:
  - Accuracy (blue bar): Approximately 0.96
  - Coverage (red bar): Approximately 0.91
  - Emphasis (yellow bar): Approximately 0.96
- **Personalized Examples**:
  - Accuracy (blue bar): Approximately 0.92
  - Coverage (red bar): Approximately 0.88
  - Emphasis (yellow bar): Approximately 0.94
- **QuizMe**:
  - Accuracy (blue bar): Approximately 0.97
  - Coverage (red bar): Approximately 0.96
  - Emphasis (yellow bar): Approximately 0.98
- **Slides**:
  - Accuracy (blue bar): Approximately 0.95
  - Coverage (red bar): Approximately 0.93
  - Emphasis (yellow bar): Approximately 0.96
- **Audio Lesson**:
  - Accuracy (blue bar): Approximately 0.99
  - Coverage (red bar): Approximately 0.92
  - Emphasis (yellow bar): Approximately 0.96
- **Mindmap**:
  - Accuracy (blue bar): Approximately 0.92
  - Coverage (red bar): Approximately 0.87
  - Emphasis (yellow bar): Approximately 0.96
- **Video**:
  - Accuracy (blue bar): Approximately 0.96
  - Coverage (red bar): Approximately 0.95
  - Emphasis (yellow bar): Approximately 0.95
- **Overall Experience**:
  - Accuracy (blue bar): Approximately 0.98
  - Coverage (red bar): Approximately 0.95
  - Emphasis (yellow bar): Approximately 0.97

## **Source Materials**

The PDFs used as the souce-of-truth for the pedagogical evaluations were all thanks to OpenStax. The 10 varied PDF used are listed in the table below:

*
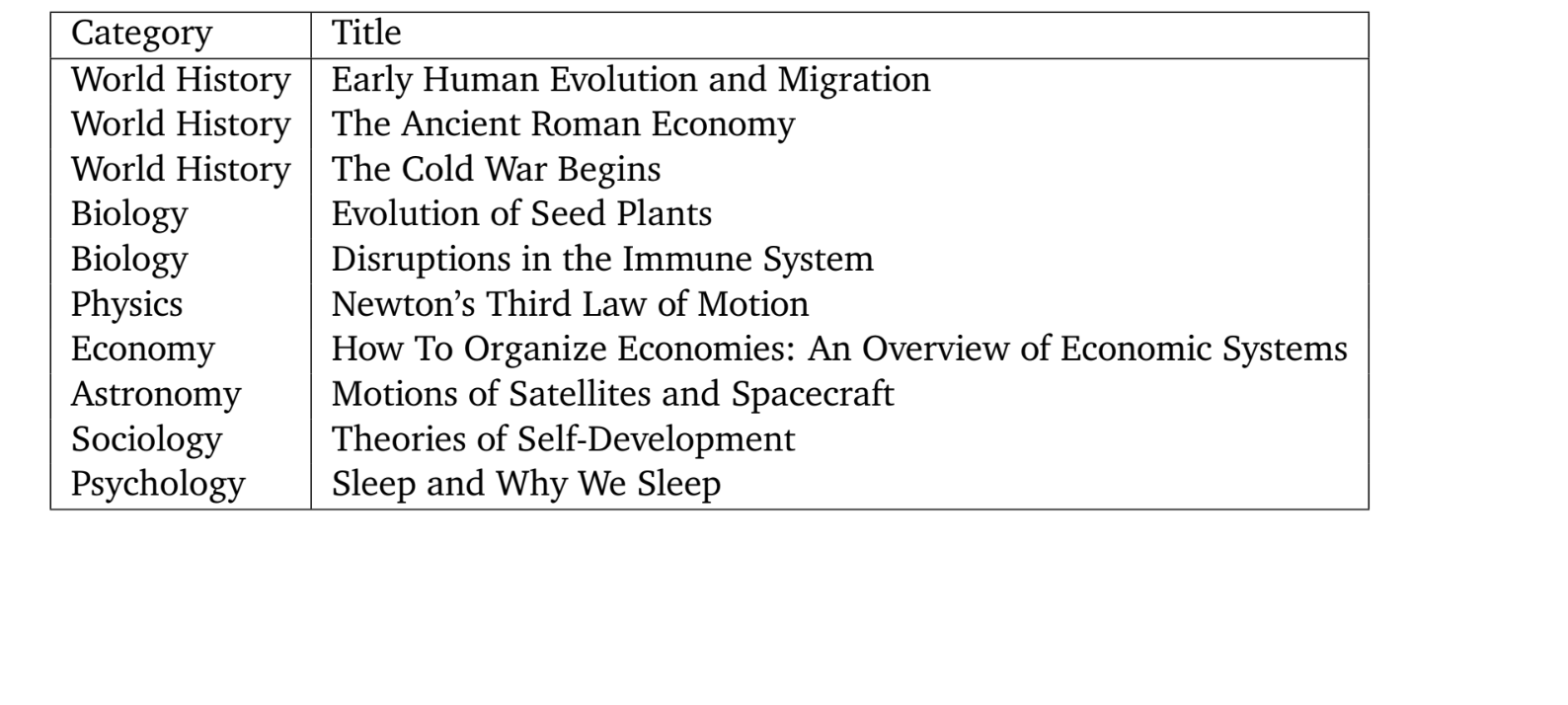
*
